# Getting Through the Door: Lessons Learned from Recruiting Assisted Living Facilities for Group Interventions

**DOI:** 10.1093/geroni/igaf122.4253

**Published:** 2025-12-31

**Authors:** Chirantana Dayanandaswamy

**Affiliations:** University of South Florida GEC, Tampa, Florida, United States

## Abstract

Engaging assisted living facilities (ALFs) is critical for implementing non-pharmacological social interventions that support residents with dementia. This study reports recruitment outcomes and lessons learned from an 8-week group music intervention conducted in Hillsborough County, Florida, from August 2024 to July 2025. A total of 166 licensed ALFs were identified, with 54 (32.5%) meeting eligibility criteria based on a minimum bed capacity of 20. Facility contact information was collected manually from their websites, and outreach was prioritized by facility size. Recruitment methods included phone calls, email follow-ups, in-person meetings, attendance at facility events, flyer distribution, and professional referrals. For facilities expressing interest by phone, our team visited the sites to conduct informational sessions and review study details. Of the 12 facilities visited, 9 (75%) agreed to start the study; six formally enrolled, and three are in the enrollment phase. Four enrolled facilities completed the intervention, while two withdrew due to staffing limitations. Across enrolled sites, 66 residents and 10 staff participated. Direct phone outreach followed by in-person visits was the most effective recruitment strategy, helping to build trust and address logistical questions. In contrast, email outreach proved ineffective, generating little to no response. Consistent, professional communication throughout the process was essential to maintaining engagement and supporting successful site participation. Our findings emphasize that constant communication, direct engagement through phone and in-person visits, and consistent professional interaction are crucial for improving recruitment and retention. These lessons can inform future efforts to implement and expand group social interventions across diverse care settings.

